# Raising Our Voices against Nuclear War: Japanese Doctors and Journals’ Roles

**DOI:** 10.31662/jmaj.2023-0141

**Published:** 2023-11-16

**Authors:** Shigeki Matsubara

**Affiliations:** 1 Department of Obstetrics and Gynecology, Jichi Medical University, Tochigi, Japan; 2 Department of Obstetrics and Gynecology, Koga Red Cross Hospital, Koga, Japan

**Keywords:** atomic bomb, editorial, Hiroshima, Nagasaki, nuclear war

## Abstract

The editorial “Reducing the risks of nuclear war - The role of health professionals” was published at the same time in 137 journals worldwide. No Japanese journals have published this editorial. Japan suffered the Hiroshima-Nagasaki atomic-bomb disaster and thus may have a special and leading position regarding this issue in the world. I believe that now is the time for us, Japanese health professionals, to raise our voices against nuclear war.

The threat of nuclear war is increasing. In August 2023, an editorial entitled: “Reducing the risks of nuclear war - The role of health professionals” was published in 137 medical journals worldwide ^(1)^. This editorial states the following: 1) the risk of nuclear war is now rapidly growing, 2) nuclear war will kill or severely injure 120 million-6 billion people, 3) health professionals played crucial roles in preventing nuclear war in history (including ending the Cold War), and 4) health-professional associations and members should take actions to prevent nuclear war now ^(1)^.

I was astounded to find that as many as 137 journals published this editorial at the same time ^(1), (2)^; these journals included the so-called big four (*New England Journal of Medicine*, the *Lancet*, *JAMA*, and *BMJ*) and also journals of all related fields: general medicine, medicine of some specialties and subspecialties, nursing, basic medicine, and others. They also included journals from many countries worldwide: those of high and middle/low resources and those from Europe, America, Africa, and Asia. Based on my 4-decades of medical writing experience, this is the first, or at least a very rare, phenomenon: therefore, many medical journals with a variety of backgrounds published the same editorials. This greatly encourages me: I felt that I am in solidarity with worldwide health professionals in terms of being against nuclear war.

The *New England Journal of Medicine* and the *Lancet* are from the USA and UK, respectively. *The Romanian Journal of Clinical Research* (among the 137 journals) is naturally from Romania. It is sometimes difficult to determine which country a certain journal is from, and thus, I checked the homepage of the 137 journals ^(2)^ and identified the institutes with which the Editor-in-Chiefs are affiliated. Based on my investigation, unfortunately, no Editor-in-Chiefs were affiliated with Japanese institutes, thus indicating that no journals were from Japan.

Japan is a unique country in the world: we, Japanese, have experienced atomic bombs. Many Japanese people still suffer sequelae of Hiroshima-Nagasaki atomic bombs physically and mentally. Japanese researchers have performed many high-level studies regarding the health problems caused by the atomic bombs ^(3), (4)^. Also, regardless of who (Japanese or non-Japanese) wrote the papers, Hiroshima-Nagasaki survivors have been continuing to provide atomic bomb-related health data to the world ^(5)^.

The lack of publishing this editorial in Japanese journals has discouraged me and possibly also many Japanese doctors. It is expected that this has also discouraged members/readers of worldwide journals outside of Japan. They may wonder why Japanese journals, and thus, Japanese health professionals, did not add their voices. I believe that *JMA Journal* should have published this editorial. I never intend to say something against the *JMA Journal*, but we, Japanese doctors, consider that this journal represents the voices of Japanese health professionals. The *JMA Journal* may have not been notified about this editorial and simply may have had no chance to be among the publishing journals.

Putting this aside, we, Japanese doctors, should raise our voices against nuclear war. This is because Japanese have experienced atomic bomb, and Japan has accumulated associated medical/social/political knowledge ^(3), (4)^, thereby having hitherto contributed to preventing nuclear war. We can educate young generations about the health-related problems caused by the atomic bombs. We can urge policy-making persons to take actions to prevent nuclear wars. I believe that the first step is: each Japanese doctor should begin such activities in his/her own position, manner, and daily medical activity. This can be, and should be, done irrespective of the personal political belief/standpoint. This might be a small step, but it may contribute to finally eradicating nuclear wars. Now is the time. I expect the continuing leadership of the *JMA Journal*.

## Article Information

### Conflicts of Interest

None

### Acknowledgement

I thank Teppei Matsubara (Massachusetts General Hospital, Harvard Medical School, USA) and Daisuke Matsubara (Jichi Medical University, Japan) for their help.

### Author Contributions

S. Matsubara: Identification of the significance. Manuscript writing.

### Approval by Institutional Review Board (IRB)

Not applicable

### Patient Anonymity

Not applicable

### Informed Consent

Not applicable

### Data Availability

Data sharing is not applicable to this article, as no new data were created or analyzed in this study.
